# Neisseria Gonorrhoeae Spontaneous Bacterial Peritonitis

**DOI:** 10.1155/2021/8865339

**Published:** 2021-03-12

**Authors:** George Mwandia, Ryan Q. Simon, Hari Polenakovik, Katelyn J. Booher

**Affiliations:** Wright State University Boonshoft School of Medicine, Department of Infectious Diseases, Weber CHE Building, 2nd Floor E Apple St. Dayton, Dayton, OH 45409-2902, USA

## Abstract

We describe a case of gonococcal spontaneous bacterial peritonitis (SBP) in a 48-year-old sexually active female with alcoholic cirrhosis and chronic hepatitis B. She was admitted with fever, abdominal pain and distension without dysuria, dyspareunia, or vaginal discharge. On exam, she was icteric with features of sepsis and tense ascites. She underwent paracentesis. The ascitic fluid analysis revealed a neutrophil count of 1,050/*µ*L, and culture grew Neisseria gonorrhoeae. Pelvic examination findings were negative for pelvic inflammatory disease; however, an endocervical swab was positive for N. gonorrhoeae by PCR. She was diagnosed with spontaneous bacterial peritonitis secondary to N. gonorrhoeae and was successfully treated with a seven-day course of IV ceftriaxone. N. gonorrhoeae spontaneous bacterial peritonitis is an extremely rare entity reported only twice despite the high prevalence of gonorrhoeae in the general population. We hypothesize that gonococcal SBP may be frequently undiagnosed since it responds to empiric antibiotics used to treat SBP. It is important for the clinician to be aware of gonococcus as a rare but potential pathogen in SBP. Future studies are needed to determine if routine gonococcal screening in SBP cases would be of clinical utility.

## 1. Objective

We hereby describe a case of gonococcal spontaneous bacterial peritonitis (SBP), an extremely rare phenomenon that to the best of our knowledge has only been described twice before.

## 2. Case Report

The patient is a 48-year-old sexually active female with decompensated cirrhosis secondary to alcohol and chronic hepatitis B on entecavir, hypertension, coronary artery disease, and hyperlipidemia. She was admitted with a one-week history of progressively worsening abdominal pain and distension associated with fevers and chills. There was no dysuria, frequency, dyspareunia, vaginal discharge, or history of sexually transmitted infection. At admission, she was icteric without asterixis or features of encephalopathy. Abnormal vital signs included tachycardia (a heart rate of 120/min) and tachypnea (22 breaths per minute). Her abdomen was distended with mild diffuse tenderness and a positive fluid wave consistent with ascites. Notable laboratory results were as follows: WBC 19.8/*µ*L, Hb 9.4 g/dL, Na 127 mmol/L, K 5.4 mmol/L, INR 1.7, BUN 71 mg/dL, creatinine 1.7 mg/dL, ammonia 77 *µ*mol/L, T. Bili 3.8 *µ*mol/L, ALP 158 U/L, AST 56 U/L, and ALT 23 U/L. Computed tomography imaging of the abdomen and pelvis revealed cirrhotic liver with extensive varices, large ascites, and splenomegaly. She underwent a 3-liter diagnostic and therapeutic paracentesis of a straw colored ascitic fluid. The ascitic fluid analysis revealed WBC 1,180/*µ*L (neutrophils 89% (an absolute neutrophil count of 1,050/*µ*L) and lymphocytes 11%) RBC 575/*µ*L, glucose 29 mg/dL, total protein 2.6 g/dL, and albumin 1.2 g/dL. Serum ascitic fluid albumen gradient (SAAG) was 0.8. Ascitic fluid culture grew Neisseria gonorrhoeae. Blood cultures yielded no growth. Pelvic examination findings were negative for pelvic inflammatory disease; however, an endocervical swab was positive for N. gonorrhoeae (and negative for *Chlamydia trachomatis*) by PCR. Wet prep and potassium hydroxide preparations were negative. She was diagnosed with SBP secondary to N. gonorrhoeae and was successfully treated with a seven-day course of IV ceftriaxone. At a 6-month follow up, she had no recurrence of her SBP or other infection.

## 3. Discussion

SBP is the most frequent bacterial infection in patients with cirrhosis. It is defined as an infection of the ascitic fluid associated with a positive bacterial culture and a polymorphonuclear cell count of at least 250/*µ*L without a surgically treatable intra-abdominal source of infection [1, 2]. When the ascitic fluid culture is negative, this entity is named “culture negative neutrocytic ascites” (CNNA) [1, 2]. The etiology of SBP is predominantly enteric bacteria, especially gram-negative bacteria such as *Escherichia coli* and Klebsiella spp. Recent data however show an increasing prevalence of gram-positive, quinolone-resistant, and multidrug-resistant bacteria [1, 3]. SBP is a cause of significant in-hospital mortality although the mortality rate has declined from 80 to 90% in the 1970 s to about 37% more recently [1, 4, 5]. SBP secondary to N. gonorrhoeae is an extremely rare entity despite the high prevalence of gonorrhea in the general population. To date, it has been reported only twice. Stassen et al. described the first case in a sexually active woman with Laennec's cirrhosis and asymptomatic cervical gonococcal colonization in 1985 [6]. Akahane et al. reported the second case in 2001. The patient was a 39-year-old Japanese female who was admitted for a fever and abdominal pain. She underwent abdominal surgery, and the ascitic fluid grew N. gonorrhoeae [7]. The mechanism of gonococcal SBP is unclear, but it is hypothesized to be transfallopian translocation of N. gonorrhoeae into the peritoneum [6]. Given the positive gonococcal endocervical probe result in our patient, it can be deduced the ascitic fluid was infected via a transfallopian route in the setting of asymptomatic gonococcal endocervicitis. Hematogenous seeding seems less likely as her blood cultures yielded no growth, and there were no other extragenital manifestations of N. gonorrhoeae infection. This scenario raises a question of whether a gonococcal screen should be part of the workup in sexually active female patients with SBP. Gonococcal infections are typically symptomatic among men, which prompts them to seek treatment. Among women, gonococcal infections are commonly asymptomatic until complications (such as pelvic inflammatory disease) occur. Does this imply that a subset of sexually active female patients with a clinical presentation of gonococcal SBP go undiagnosed as they clinically respond to antimicrobial agents used to treat spontaneous bacterial peritonitis? Could some of the cases of CNNA be caused by N. gonorrhoeae in this population of patients? Despite the foregoing, the addition of targeted gonococcal screening in all cases of female SBP patients is of unknown clinical utility.

## Data Availability

The data used to support the findings of this case report are not freely available and accessible for legal and ethical concerns and patient privacy and also because it would be a breach of patient confidentiality.
